# Higher normal ranges of urine albumin-to-creatinine ratio are independently associated with carotid intima-media thickness

**DOI:** 10.1186/1475-2840-11-112

**Published:** 2012-09-21

**Authors:** Sun-Seog Kweon, Min-Ho Shin, Young-Hoon Lee, Jin-Su Choi, Hae-Sung Nam, Kyeong-Soo Park, Do-Hyung Kim, Seul-Ki Jeong

**Affiliations:** 1 Department of Preventive Medicine, Chonnam National University Medical School, Gwangju, South Korea; 2 Department of Preventive Medicine & Institute of Wonkwang Medical Science, Wonkwang University College of Medicine, Iksan, Jeonbuk, South Korea; 3 Department of Preventive Medicine, Chungnam National University College of Medicine, Daejeon, South Korea; 4 Department of Preventive Medicine, Seonam University College of Medicine, Namwon, Jeonbuk, South Korea; 5 Department of Neurology & Research Institute of Clinical Medicine, Chonbuk National University - Biomedical Research Institute of Chonbuk National University Hospital, San 2-20, Geumam-dong, Deokjin-gu, Jeonju, Jeonbuk, 561-180, South Korea

**Keywords:** Albuminuria, Atherosclerosis, Carotid intima-media thickness, Urine albumin-to-creatinine ratio

## Abstract

**Background:**

High normal values of urine albumin-to-creatinine ratio (UACR) have been reported to have predictive values for hypertension, incident stroke, and higher mortality in the general population. This study aimed to investigate the association between normal ranges of UACR and carotid intima-media thickness (CIMT) in adult population.

**Methods:**

We performed a cross-sectional study in adults aged 45 to 74 years who were living in Namwon City, South Korea. Both common CIMTs were measured, and mean values were calculated. Normal values of UACR were defined as <30mg/g and categorized into quintiles; less than 6.50, 6.51-9.79, 9.80-13.49, 13.50-18.89, and more than 18.90 mg/g. The association between the quintiles of UACR and common CIMT was analyzed and stratified by sex.

**Results:**

A total of 7555 participants (3084 men and 4471 women) with normal UACR were enrolled in the present study. Common CIMT was positively and independently associated with increasing quintiles of UACR in men and women, even after adjusting for potential confounders including age and cardiovascular risk factors. Compared to the first quintile, the fifth quintile showed odds ratios of 1.80 (95% confidence intervals, 1.26-2.55) and 1.97 (1.28-3.04) for increased CIMT (>0.9mm) in men and women, respectively.

**Conclusion:**

Higher UACR values within normal ranges (<30 mg/g) were positively and independently associated with CIMT in a Korean general population, suggesting that higher normal values of UACR might be a risk marker of subclinical carotid atherosclerosis.

## Introduction

Atherosclerosis and its associated vascular effects including cardiovascular disease (CVD), stroke, peripheral arterial disease, and end-stage renal disease has become a leading cause of disability and mortality in both developed and developing countries [1]. For an effective prevention of vascular diseases, it would be reasonable to discuss the limitations of conventional risk equations due to the increase in obesity, metabolic syndrome, and type 2 diabetes. And it needs to be developed for biomarkers that are relevant to the risk in those individuals [2].

Elevations in albumin excretion are indicative of glomerular injury mainly due to type 2 diabetes or hypertension [3]. Rather than using 24-hr urine collections, spot urine tests can determine urinary albumin-to-creatinine ratios (UACR), making it easier to diagnose and monitor albuminuria both clinically and in research studies [4]. In addition to renal injury, albuminuria (usually defined as UACR ≥ 30mg/g) has become a biologic marker of underlying systemic diseases such as atherosclerosis [5]. Albuminuria has also been associated with incident strokes in various populations [6] and in both diabetics and non-diabetics [7]. High normal values of UACR predicted hypertension [8], incident stroke [9], and higher mortality [10] in the general population.

Albuminuria has been associated with carotid intima-media thickness (CIMT) [11,12], which is a widely accepted marker of subclinical CVD [13]. Similarly, the association of normal range UACR values (i.e., <30mg/g) and CIMT should be studied separately, because the normal ranges of UACR has been reported to have predictive values for subsequent hypertension and mortality [8,10]. In this cross-sectional study, we reported the association between normal UACR values (<30mg/g) and CIMT in an adult Korean population aged 45 to 74 years. To determine the independence of the above association, data were adjusted for cardiovascular risk factors and estimated glomerular filtration rate (eGFR), which showed a significant linear relationship with UACR values when stratified by sex.

## Subjects and methods

### Study population

The Namwon study is an ongoing prospective study designed to investigate the prevalence, incidence, and risk factors for chronic disease in Namwon City, Chonbuk province, Korea. Demographic data and measurements for the study subjects were as previously reported [14]. The 2005 census reported 33,068 residents (14,960 men and 18,108 women) aged 45–74 in Namwon City. From 2004 to 2007, all eligible residents within this age range were contacted via mail or telephone and were invited to participate in the study. A total of 10,667 subjects (4,201 men and 6,466 women; response rate 32.3%) chose to participate in the clinical examinations and interviews. This study was approved by the institutionalethics committee at Chonbuk National University Hospital, and all participating subjects provided written informed consent.

### Assessments and measurements

The clinical data collected from the subjects included prior history of CVD, CVD risk factors, and medication usage. Major cardiovascular risk factors included hypertension (blood pressure ≥140/90mmHg or use of anti-hypertensive medication), type 2 diabetes (current or previous use of glucose-lowering medications or fasting blood glucose >7.0mmol/L (126mg/dL)), and hyperlipidemia (previous use of lipid-lowering medications, total cholesterol ≥6.2mmol/L (240mg/dL), or low density lipoprotein (LDL) cholesterol ≥4.1mmol/L (160mg/dL)). Smoking status was divided into 3 categories: current smokers, former smokers, and nonsmokers. Participants also answered questions concerning alcohol consumption, including questions about frequency, duration, amount, and type of alcohol consumed. Waist circumference was measured at the point midway between the lower rib margin and the iliac crest with the subject standing. Body mass index (BMI) was calculated as weight divided by height squared (kg/m^2^).

Blood pressure was measured on the right arm with a standard mercury sphygmomanometer after 5min of rest in the sitting position. The appearance (phase I) and disappearance (phase V) of Korotkoff sounds were used to define systolic and diastolic pressure, respectively. Blood pressure readings were recorded to the nearest even number, and three readings were taken at 1 min intervals from each participant. The mean value of the three readings was considered to represent the individual’s blood pressure.

Blood samples were drawn after a 12-hour overnight fasting period, and standard techniques were used to measure serum chemistries and lipids. Serum insulin was determined by immunoassay using AxSYM (Abbott Laboratories, IL, USA), and insulin resistance was calculated using the homeostasis model assessment of the insulin resistance (HOMA-IR) score [15]. Each participant provided an early morning spot urine sample in a sterile container at the examination center. Urinary albumin concentrations were measured using a turbidimetric immunoassay, and creatinine concentrations were measured using the Jaffe method on an automated analyzer (Hitachi-7600, Hitachi, Ltd). All laboratory procedures were conducted at the same laboratory. The UACR was defined as the urinary albumin value divided by the urinary creatinine concentration (mg/g). The eGFR was calculated using the 6-variable Modification of Diet in Renal Disease (MDRD) equation [16]:

(none) $$\begin{array}{cc} \text{eGFR} & =170\times {\text{serum creatinine}}^{-0.999}\times {\text{age}}^{-0.176}\\ & \;\times {\text{BUN}}^{-0.170}\times {\text{albumin}}^{0.318}\\ & \;\left(\times 0.762\;\text{if the participant was a woman}\right)\text{.} \end{array}$$

The eGFR is expressed in mL/min/1.73m^2^, serum creatinine and blood urea nitrogen (BUN) are expressed in mg/dL, albumin is expressed in g/dL, and age is expressed in years. Abnormal kidney function was defined as eGFR <60mL/min/1.73m^2^.

Details of carotid intima-media thickness (CIMT) measurements are as previously published [17]. Distal portions of the common carotid artery (CCA) IMT were assessed by two certified physicians (S.K.J. and Y.H.L.) using high-resolution B mode ultrasonography (SONOACE 9900, Medison, Korea) with an electrical linear array transducer (7.5MHz). Images of plaque free regions as well as the thickest areas (>10mm) from the carotid bulb to the CCA were saved and measured using SigmaScan Pro Version 5.0.0 (SPSS Inc., Chicago, IL, USA). CIMT thickening was defined as a mean CIMT of ≥0.9mm. The correlation coefficients for inter- and intra-examiner variability for 189 subjects were 0.86 and 0.90, respectively.

### Statistical analysis

Data are expressed as mean ± standard deviation (SD) or percentages. Students t-tests were used to analyze differences between continuous variables, and chi-square tests were used for categorical variables. According to the UACR quintile values, means and proportions of CVD risk factors were calculated using analysis of variance (ANOVA) or chi-square tests. All analyses were performed after sex stratification. Analysis of covariance (ANCOVA) was used to evaluate the association between common CIMT and UACR quintile values. Logistic regression analysis was also used to evaluate the association between the UACR quintile values and CIMT thickening, which are presented as odds ratios (OR) and 95% confidence intervals. All statistical analyses were conducted using PASW statistics 18 (SPSS Inc., Chicago, IL, USA).

## Results

Among the 10,667 subjects, 7847 (73.6%) were defined as having UACR values <30mg/g, 2687 (25.2%) had values >30mg/g, and 133 (1.2%) had unmeasured UACRs. Among the subjects with a UACR <30mg/g, 292 (3.7%) did not complete subsequent testing including carotid ultrasonography or blood chemistry. A total of 7,555 subjects (70.8%; men: 3,084, women: 4,471) with UACR values <30mg/g who underwent carotid ultrasonography were analyzed in the present study*.*

The demographic and clinical features of the study population are presented in Tables 1 and 2 in men and women, respectively, because all CVD risk factors showed significant differences between them. Nearly all CVD risk factors showed significant positive associations with UACR values in men and women, including age, hypertension, type 2 diabetes, systolic BP, diastolic BP, triglycerides, fasting blood sugar, serum creatinine, and eGFR. On the other hand, proportions of current smokers, hyperlipidemia, BMI, total cholesterol, and HDL-cholesterol did not show significant associations with the UACR values. Proportions of current drinkers, waist circumference, and HOMA-IR were significantly and positively associated with UACR values only in men.

**Table 1:** Cardiovascular risk profiles according to quintiles of UACR in men

	**UACR, mg/g**					p^*^
	**< 6.50**	**6.5-9.79**	**9.80-13.49**	**13.50-18.89**	**≥ 18.90**	
Number (%)	691 (22.4)	692 (22.4)	641 (20.8)	549 (17.8)	511 (16.6)	
Age, y	60.2 ± 8.0	61.0 ± 7.9	62.3 ± 7.4	63.4 ± 7.3	63.4 ± 7.2	< 0.001
Current smoker, %	29.8	35.0	39.6	33.4	33.1	0.287
Current drinker, %	63.6	62.4	66.7	66.9	67.5	0.043
Hypertension, %	31.8	33.7	33.0	41.2	47.0	< 0.001
Type 2 diabetes, %	8.0	8.7	9.8	13.5	16.3	< 0.001
Hyperlipidemia, %	6.5	6.1	7.2	6.7	7.8	0.331
Body mass index, kg/m^2^	24.0 ± 2.8	23.7 ± 2.7	23.8 ± 2.7	23.8 ± 2.9	24.1 ± 2.8	0.059
Waist circumference, cm	85.5 ± 7.6	84.4 ± 7.3	85.1 ± 7.8	84.7 ± 8.2	86.0 ± 7.8	0.004
Systolic BP, mmHg	121.1 ± 15.9	122.3 ± 15.1	124.3 ± 15.2	127.7 ± 16.2	129.6 ± 15.9	< 0.001
Diastolic BP, mmHg	79.6 ± 9.3	79.6 ± 9.6	79.9 ± 9.3	81.7 ± 9.9	82.1 ± 10.2	< 0.001
Total cholesterol, mmol/L	4.7 ± 0.9	4.7 ± 0.9	4.7 ± 0.9	4.7 ± 0.9	4.8 ± 1.0	0.246
Triglycerides, mmol/L	1.8 ± 1.4	1.7 ± 1.3	1.8 ± 1.3	1.8 ± 1.3	2.0 ± 1.4	< 0.001
HDL cholesterol, mmol/L	1.2 ± 0.3	1.2 ± 0.3	1.2 ± 0.3	1.2 ± 0.3	1.2 ± 0.3	0.727
FBG, mmol/L	5.7 ± 0.9	5.7 ± 0.9	5.7 ± 1.0	5.8 ± 1.1	6.1 ± 1.6	< 0.001
HOMA-IR	1.4 ± 1.3	1.2 ± 1.1	1.3 ± 1.4	1.2 ± 1.0	1.6 ± 1.9	< 0.001
Serum creatinine, μmol/L	91.5 ± 16.7	87.9 ± 13.3	87.4 ± 13.1	86.0 ± 13.6	86.9 ± 13.6	< 0.001
eGFR, ml/min/1.73m^2^	83.9 ± 13.0	86.9 ± 14.3	87.0 ± 14.0	88.5 ± 15.4	87.1 ± 14.1	< 0.001

Data are expressed as mean ± standard deviation, unless otherwise indicated. BP, blood pressure; HDL; high-density lipoprotein, FBG, fasting blood glucose; HOMA-IR; homeostatic model assessment for insulin resistance, eGFR; estimated glomerular filtration rate.

eGFR was calculated using the 6-variable Modification of Diet in Renal Disease (MDRD) formula.

^*^ p-values for analysis of variance (ANOVA) and chi-square tests for trends are shown, as appropriate.

**Table 2:** Cardiovascular risk profiles according to quintiles of UACR in women

	**UACR, mg/g**					p^*^
	**< 6.50**	**6.5-9.79**	**9.80-13.49**	**13.50-18.89**	**≥ 18.90**	
Number (%)	798 (17.8)	839 (18.8)	882 (19.7)	928 (20.8)	1024 (22.9)	
Age, y	56.8 ± 7.8	58.8 ± 7.9	60.2 ± 7.7	62.0 ± 7.3	62.9 ± 7.3	< 0.001
Current smoker, %	3.4	4.5	2.3	4.0	2.8	0.371
Current drinker, %	38.8	41.1	34.8	35.0	36.8	0.054
Hypertension, %	24.8	27.7	31.6	33.5	42.9	< 0.001
Type 2 diabetes, %	5.1	5.5	7.2	8.3	9.8	< 0.001
Hyperlipidemia, %	11.8	11.5	11.1	12.9	12.4	0.441
Body mass index, kg/m^2^	24.5 ± 3.0	24.6 ± 3.0	24.6 ± 3.2	24.5 ± 3.1	24.5 ± 3.2	0.969
Waist circumference, cm	86.4 ± 7.9	86.4 ± 8.4	86.4 ± 8.7	86.1 ± 8.8	86.0 ± 8.9	0.714
Systolic BP, mmHg	117.8 ± 16.0	119.6 ± 16.0	121.5 ± 16.7	123.6 ± 17.2	127.2 ± 18.6	< 0.001
Diastolic BP, mmHg	77.2 ± 9.4	77.4 ± 9.0	78.5 ± 9.4	79.4 ± 9.5	80.6 ± 10.8	< 0.001
Total cholesterol, mmol/L	4.9 ± 1.0	4.9 ± 0.9	5.0 ± 0.9	5.0 ± 0.9	5.0 ± 1.0	0.139
Triglycerides, mmol/L	1.5 ± 1.0	1.5 ± 1.0	1.6 ± 1.0	1.7 ± 1.0	1.7 ± 1.4	< 0.001
HDL cholesterol, mmol/L	1.3 ± 0.3	1.3 ± 0.3	1.2 ± 0.3	1.2 ± 0.3	1.3 ± 0.3	0.400
FBG, mmol/L	5.5 ± 0.7	5.5 ± 0.8	5.5 ± 0.8	5.5 ± 1.0	5.6 ± 1.0	0.001
HOMA-IR	1.4 ± 0.9	1.4 ± 1.3	1.4 ± 1.1	1.4 ± 1.1	1.4 ± 1.2	0.449
Serum creatinine, μmol/L	70.8 ± 9.6	70.2 ± 10.1	68.6 ± 11.5	67.4 ± 9.6	68.1 ± 10.9	< 0.001
eGFR, ml/min/1.73m^2^	83.8 ± 12.7	84.1 ± 13.0	86.0 ± 14.9	86.2 ± 13.3	85.6 ± 14.5	< 0.001

Data are expressed as mean ± standard deviation, unless otherwise indicated.

^*^ p-values for analysis of variance (ANOVA) and chi-square tests for trends are shown, as appropriate.

**Figure 1:**
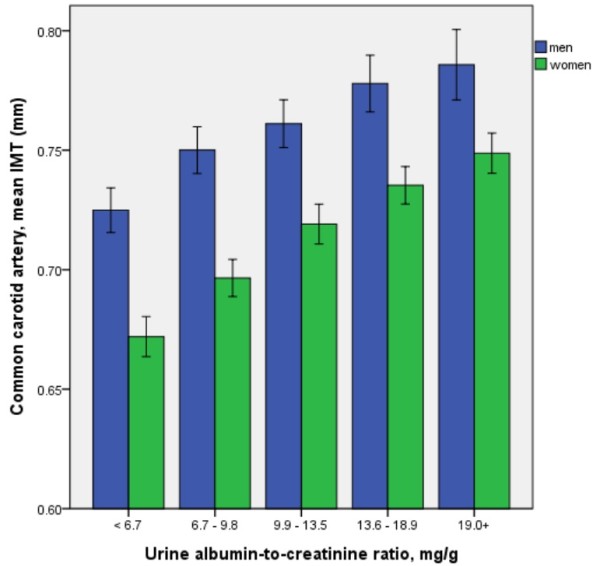
**The mean values of common carotid intima-media thickness (CIMT) according to quintiles of normal UACR values in men and women (both p values < 0.001 by analysis of variance, respectively).** Error bars indicate 95% confidence intervals.

Higher UACR values within normal ranges were significantly and positively associated with distal CIMT in men and women, as depicted in Figure 1. The mean value of CIMT was significantly higher in men than women in each quintile (all p values < 0.001). The associations between higher UACR values within normal ranges and CIMT in men and women were independent, even after adjusting for age, current smoking status, current alcohol use, waist circumference, systolic BP, diastolic BP, HOMA-IR, eGFR, triglycerides, fasting blood glucose, hypertension, type 2 diabetes, and hyperlipidemia as shown in Table 3. UACR values were also independently associated with CIMT thickening (>0.9 mm) in men and women, as shown in Table 4.

**Table 3:** Adjusted mean values of common carotid IMT according to quintiles of UACR (<30mg/g)

	**Adjusted carotid IMTs (Mean ± SE, mm) according to quintiles of UACR (mg/g)**					**p for overall**	**p for linear**
**Sex**	< 6.50	6.5-9.79	9.80-13.49	13.50-18.89	≥ 18.90	**difference**	**trend**
Men	0.742 ± 0.005	0.753 ± 0.005	0.761 ± 0.005	0.764 ± 0.005	0.769 ± 0.006	0.003	< 0.001
Women	0.701 ± 0.004	0.711 ± 0.004	0.721 ± 0.004	0.722 ± 0.004	0.727 ± 0.004	< 0.001	< 0.001

Data are expressed as mean ± standard error.

Adjusted for age, current smoking status, current alcohol use, waist circumference, systolic BP, diastolic BP, HOMA-IR, eGFR, triglycerides, fasting blood glucose, hypertension, type 2 diabetes, and hyperlipidemia.

**Table 4:** Associations between carotid IMT thickening (>0.9 mm) and quintiles of UACR

**Quintiles of UACR, mg/g**	**Odds ratio (95% confidence intervals)**	
		**Men**	**Women**
< 6.50	1.00	1.00
6.5-9.79	1.33 (0.94-1.89)	1.14 (0.70-1.88)
9.80-13.49	1.39 (0.99-1.97)	1.82 (1.16-2.86)^**^
13.50-18.89	1.63 (1.15-2.55)^**^	1.61 (1.03-2.52)^*^
≥ 18.90	1.80 (1.26-2.55)^**†^	1.97 (1.28-3.04)^**†^

Adjusted for age, current smoking status, current alcohol use, waist circumference, systolic BP, diastolic BP, HOMA-IR, eGFR, triglycerides, fasting blood glucose, hypertension, type 2 diabetes, and hyperlipidemia.

^*^ p < 0.05, ^**^ p < 0.01.

^†^ p < 0.001 by trend test.

## Discussion

Our results demonstrated that common CIMT was positively associated with UACR values in a Korean population aged 45 to 74 years. The UACR values were limited to less than the recommended cutoff value (30mg/g), and the associations were adjusted for age and CVD risk factors in both sexes. UACR values >30mg/g are indicative of renal injury, and kidney disease is an independent risk factor for the occurrence of vascular diseases [18]. Thus, UACR values less than 30mg/g are likely better for assessing associations with CIMT and CVD risk factors to exclude the confounding effects of kidney disease. No previous study has reported the cross-sectional association between the UACR values of normal range and CIMT in detail. There have been two previous studies that showed the similar results, but one study included subjects with microalbuminuria [19] and the other showed the UACR values according to CIMT thickening only [20].

### Carotid IMT and higher UACR values within normal ranges

The significant association between CIMT and higher UACR values within normal ranges (<30mg/g) are likely the result of systemic atherosclerosis, which presents differently depending on the organ system. In the kidney, an increase in the UACR represents a loss of podocytes and an elevation in angiotensin II [21], while CIMT thickening is a surrogate marker of macroangiopathy [22]. The etiology of increases in both UACR and common CIMT is thought to be endothelial dysfunction [23,24].

Both the UACR values and CIMT respond similarly to various therapeutic treatments. Carotid atherosclerosis has been shown to improve with the use of antihypertensives [25], antiplatelet agents [26], and intensive diabetes management [11]. UACR values may also be lowered with the use of antihypertensives [27], antidiabetics [28], and exercise [29]. Treatment with angiotensin II blockade has shown that carotid atherosclerosis and UACR values can improve simultaneously [30], suggesting that angiotensin II levels may play a role in elevated UACR values and CIMT.

In the present study, men showed significantly higher CIMT than women in each quintile of UACR. The similar findings were also observed in diabetic patients, and the mean annual progression of CIMT was associated with age only in men [31]. However, the 10-year risk prediction of first-time myocardial infarction or stroke was not different in men and women with an addition of CIMT to the Framingham Risk Score, which showed small improvement of limited clinical importance [32].

### UACR values and cardiocerebrovascular disease risk factors

Nearly all CVD risk factors showed linear relationships with UACR values, as presented in Tables 2 and 3. Among the lipid profiles, triglycerides were independently associated with the higher UACR values within normal ranges in both sexes, but total cholesterol and HDL-cholesterol were not. A recent study showed that triglycerides were significantly lowered with olmesartan [33] which supports the proposed interaction between the renin-angiotensin-aldosterone system and lipid metabolism [34].

In this study, many of the CVD risk factors studied were sex specific. In women, UACR values <30mg/g were not associated with obesity indices (BMI and waist circumference), whereas significant associations were observed in men. Obesity has been shown to be an independent risk factor for the development of chronic kidney disease as well as poor overall health outcomes [35]. It is important to note, however, that UACR values <30mg/g should be considered carefully in obese patients, as muscle mass, which determines the urinary concentration of creatinine, may be more variable in obese women than men [36]. Thus, obesity may have a confounding effect on UACR, particularly in women. Similar findings have also been reported for microalbuminuria [37].

The UACR is a window into the renal artery, which shares many features with cerebral arteries [38]. The vascular beds of the brain and kidney have similar hemodynamic properties in terms of high blood flow and low impedance with tightly autoregulated perfusion [39]. In addition to a strong and independent association with stroke, albuminuria was also independently associated with cognitive decline [40,41]. UACR levels not traditionally considered significant might be used to identify cerebral arterial dysfunction even before evident diseases, such as stroke or dementia, are clinically manifested [42].

### Limitations

There are several limitations of our study. First, due to the cross-sectional study design, a causal relationship between UACR values <30mg/g, CVD risk factors, and CIMT cannot be inferred. Second, a single measurement of UACR may not adequately characterize *in vivo* variability of this value. Although we adjusted for urinary creatinine excretion, the assessment of albumin excretion with spot urine tests may be less accurate than 24-hour urine collections. Third, there might be a potential residual confounding for the treatment of risk factors, especially with lipid-modifying therapies, to explain the discrepant associations between UACR values and lipid profiles. Finally, the response rate (32.3%) was relatively low, and the subjects were not randomly selected in our study. These might result in selection bias and limit the generalization of the present findings. Although, age and sex distribution in the study subjects were comparable with those of the general population, suggesting that the present study overcame volunteer effects in part.

## Conclusion

In a population of Korean adults, higher normal values of UACR were independently and positively associated with common CIMT, even after adjusting for potential confounders, suggesting that higher normal values of UACR might be a risk marker of subclinical carotid atherosclerosis.

## Abbreviations

UACR: Urine albumin-to-creatinine ratio; CIMT: Carotid intima-media thickness; CCA: Common carotid artery; CVD: Cardiovascular disease; EGFR: Estimated glomerular filtration rate; BMI: Body mass index; HOMA-IR: Homeostatic model assessment for insulin resistance; MDRD: Modification of diet in renal disease; BUN: Blood urea nitrogen; SD: Standard deviation; ANOVA: Analysis of variance; ANCOVA: Analysis of covariance; OR: Odds ratio; BP: Blood pressure; HDL: High density lipoprotein.

## Competing interests

The authors declare that they have no competing interests.

## Authors’ contributions

SSK, MHS, YHL, JSC, HSN, KSP, and SKJ performed the community cohort. SSK and SKJ performed all the data extraction and computation independently. SKJ wrote the paper. DHK, MHS, YHL, JSC, HSN, and KSP took part in writing the paper. All authors reviewed and agreed the final version of manuscript. All authors read and approved the final manuscript.
